# Molecular Insights into Oral Manifestations in Sjögren’s Disease

**DOI:** 10.3390/ijms27094144

**Published:** 2026-05-06

**Authors:** Konstantina Xanthopoulou, Anna Siatra, Konstantinos I. Tosios, Eleni-Marina Kalogirou

**Affiliations:** 1 School of Dentistry, National and Kapodistrian University of Athens, 11527 Athens, Greece; dinaxanth@gmail.com (K.X.); annasiatra24@gmail.com (A.S.); ktosios@dent.uoa.gr (K.I.T.); 2 Faculty of Health Sciences, Metropolitan College, 15125 Athens, Greece

**Keywords:** Sjögren’s disease, Sjögren’s syndrome, autoimmune disease, dry mouth, xerostomia, oral manifestations, saliva, salivary gland epithelial cells, immune cells, molecular pathways

## Abstract

Sjögren’s Disease is an autoimmune epithelitis targeting the exocrine glands, predominantly the salivary and lacrimal glands, resulting in the major symptoms of dry mouth and dry eyes. The aim of this study is to review the pertinent literature on studies linking the oral manifestations of SjD patients, with the underlying molecular events driving SjD pathogenesis. These include mechanisms inducing innate sensing in salivary gland epithelial cells, activation of interferon signaling pathway, amplification of cytokines and chemokines, and orchestration of the inflammatory milieu in salivary glands, as well as mechanisms inducing salivary epithelial tissue destruction and secretory dysfunction, such as programmed cell death pathways, mitochondrial dysfunction, structural disorganization, loss of junctional integrity, and quantitative and qualitative aberrations in salivary secretory process.

## 1. Introduction

Sjögren’s Disease (SjD) is an autoimmune epithelitis targeting the exocrine glands, predominantly the salivary and lacrimal glands, that progressively results in parenchymal destruction and glandular dysfunction, manifesting as dry mouth and dry eyes, respectively [1,2]. The term “disease” was recently endorsed to replace the “syndrome” nomenclature in order to emphasize that SjD is a particular autoimmune disorder, with distinct underlying pathogenesis resulting in specific glandular histopathological features, circulating autoantibodies, and characteristic systemic manifestations. Moreover, SjD exhibits clinical severity, significant morbidity, and, in some cases, increased mortality, rather than a collection of mainly sicca-related signs and symptoms [3,4]. SjD may arise as an isolated autoimmune disorder (“primary SjD”) or in association with other autoimmune diseases, such as rheumatoid arthritis, systemic lupus erythematosus, and systemic sclerosis [4].

The global prevalence of SjD is 0.01–0.05%, showing a clear female predominance, with a female-to-male ratio of 9:1 [5]. SjD usually affects middle-aged patients, most often in the 5th or 6th decade of life [5], while it is diagnosed at a younger age in African and African-American populations compared with Europeans [6]. Children are rarely diagnosed with SjD, although it is estimated to be more prevalent than recognized in childhood [7].

Beyond glandular dysfunction, SjD might present with fatigue and musculoskeletal pain, as well as hematological and/or multisystem complications, and an elevated risk for the development of non-Hodgkin lymphoma [8], which is 4.3-fold higher than that of the general population [9]. Lymphoma arises in 5–10% of SjD patients [10,11] and has been associated with the underlying chronic inflammatory milieu that promotes the sustained B-cell activation, proliferation, and, eventually, malignant transformation [12]. The most common lymphoma subtype among SjD patients is the Mucosa-Associated Lymphoid Tissue (MALT) lymphoma, which mainly involves the parotid and submandibular glands but may also develop in the nasopharynx, orbit, thyroid, lung, and stomach [11].

The diagnosis of SjD is rendered according to the American College for Rheumatology-European Alliance of Associations for Rheumatology (ACR–EULAR) criteria defined in 2016 by primarily evaluating serological results for anti-Sjögren’s syndrome type A (anti-SSA/Ro) antibody positivity and the presence of focal lymphocytic sialadenitis in the minor salivary gland biopsy, with each assigned a score of 3 [13]. In addition, ocular tests, i.e., Schirmer’s test and ocular staining score/van Bijsterveld score, as well as the measurement of the unstimulated whole salivary flow rate, which are each weighted at score of 1, are often required to achieve the minimum final score of 4 that is diagnostic for SjD [13]. Of note, over 30% of SjD patients might be negative for anti-SSA/Ro antibody in the so-called “seronegative SjD” [14]. The inclusion of two tests related to the oral cavity among the diagnostic criteria of SjD highlights the clinical significance of oral manifestations in SjD patients and, indirectly, the responsibility of clinicians involved in oral examination for the prompt recognition of the disease. SjD withholds the risk of severe extra-glandular complications and damage of vital organs and often impairs the quality of life of the affected patients because of chronic fatigue, anxiety, depression, and impaired physical activity [10,15]. Thus, early diagnosis is vital for optimizing patient outcomes and potentially minimizing any disease-related complications [10].

Xerostomia is a major and early manifestation of SjD with a detrimental effect on oral health, as well as general health, and it can be indicative of the diagnosis [16,17]. Xerostomia might be reported by patients with autoimmune diseases other than SjD, such as rheumatoid arthritis, systemic lupus erythematosus, and systemic sclerosis [18]; however, dry mouth is the predominant characteristic of SjD patients, resulting in other oral complications, such as dental caries and *Candida* infection [19]. Salivary gland enlargement might be also encountered in patients with other inflammatory, infectious, neoplastic, or systemic diseases [15]. IgG4-related sialadenitis, which is one of the most common manifestations of the IgG4-related disease, shares oral similarities with SjD, including salivary gland swelling and sicca symptoms [20,21]. Xerostomia, though, is statistically significantly more frequently observed among SjD patients than individuals with IgG4-related sialadenitis [22]. The IgG4-related sialadenitis is microscopically characterized by prominent IgG4-positive plasma cell infiltration and storiform fibrosis, in contrast to the focal periductal lymphocytic infiltration seen in the salivary glands of SjD patients [20,22]. Moreover, patients with IgG4-related sialadenitis usually present with increased serum IgG4 levels, whereas anti-SSA/Ro and anti-Sjögren’s syndrome type B (anti-SSB/La) positivity is rarely noted [20]. These differences in histopathological, serological, and clinical findings highlight the distinct pathogenesis of SjD and IgG4-related disease and facilitate the differential diagnosis between the two diseases.

Several previous reviews focused on general pathogenetic mechanisms [23,24,25,26] or epithelial cells or/and immune cells activation in the salivary glands in SjD [2,27,28,29,30,31,32], as well as on oral signs and symptoms of SjD patients, mainly from a clinical viewpoint [17,33,34,35]. The novelty of this review is that it aims to thoroughly summarize the pertinent literature on studies linking the oral manifestations of SjD patients with the underlying molecular events driving SjD pathogenesis.

## 2. Dry Mouth

Oral dryness or xerostomia is reported by over 90% of SjD patients at the time of SjD diagnosis or during the disease course [36]. The objective assessment of xerostomia is performed via measurement of the unstimulated whole salivary flow rate for over 15 min, and a flow rate of ≤0.1 mL/min is regarded as pathological [15]. Reduced salivary flow rate can lead to functional alterations, such as difficulties in speaking, eating, swallowing, and wearing dentures, and increases susceptibility to dental caries, premature tooth loss, and infections, such as candidiasis, due to loss of antimicrobial capacity of saliva [15,37]. The prolonged hyposalivation might result in several clinical signs of oral dryness, including oral mucosal atrophy, tongue erythema, and fissuring, as well as presence of residual food debris [34]. Aiming to objectively assess oral dryness, Osailan et al. [38] invented a 10-point clinical scale based on the following parameters (each rated with 1 point): dental mirror sticking to buccal mucosa, dental mirror sticking to tongue, presence of frothy saliva, absence of saliva in the floor of mouth, tongue depapillation, lobulated or fissured tongue, presence of altered/smooth gingiva, presence of glassy oral mucosa, presence of current or recently restored cervical caries in more than two teeth, and presence of debris on non-denture supporting palatal areas. A significantly higher oral dryness score has been observed in primary SjD patients compared with non-SjD xerostomic patients [38,39], as well as compared with healthy controls [39]. Tongue alterations, including atrophy of the filiform and/or fungiform lingual papillae, often combined with prominent furrows on the dorsal tongue surface, resulting in a cobblestone-like appearance, are usually observed among SjD individuals [34,35,40]. Moreover, a significantly higher rate of atrophic, fissured tongue has been observed among SjD patients with MALT lymphoma compared with non-lymphoma SjD subjects [40].

The histopathological hallmark of SjD in salivary glands is the formation of focal sialadenitis, expressed via the “focus score” parameter [41]. The focus score is described as the mean number of foci per 4 mm^2^ of salivary gland tissue, while one focus is defined as an aggregate of at least 50 lymphocytes [42]. Focus score corresponds to a semiquantitative assessment of the inflammatory infiltration severity [41] and shows a strong negative correlation with the salivary flow rate of SjD patients [43].

The decreased salivary flow rate in SjD patients is the consequence of impaired production and secretion of saliva by the salivary gland epithelial cells (SGECs), due to their destruction mediated by the chronic lymphocytic infiltration [29]. In turn, SGECs injury promotes release of autoantigens that stimulate activated T cells and B cells to establish an intense inflammatory response, resulting in exacerbation of sicca symptoms [29]. The initial pathogenic event in SjD is the interplay between the SGECs and the immune system that triggers a cascade of molecular events, including activation of the interferon (IFN)—Janus Kinases (JAK)—Signal Transducer and Activator of Transcription (STAT) axis, amplification of cytokines and chemokines, and further recruitment of immune cells. This sequence promotes the organization of the inflammatory milieu within the salivary glands that disrupts homeostasis and normal metabolism of SGECs and causes the salivary gland dysfunction that manifests clinically with dry mouth [2,27,29,44]. Thus, several randomized, double-blind, placebo-controlled, and open-label clinical studies have evaluated the therapeutic effect of targeted treatments in the unstimulated and stimulated salivary flow levels of SjD patients (Table 1).

### 2.1. Innate Sensing in SGECs

SGECs act as intrinsic immune sensors capable of promoting the initiation and the perpetuation of the localized autoimmune inflammatory response in SjD [29]. SGECs of SjD patients express immunoreactive molecules involved in antigen presentation, e.g., the major histocompatibility complex (MHC) class I and II, co-stimulation peptides (B7.1/CD80 and B7.2/CD86), and adhesion molecules, e.g., the intercellular adhesion molecule-1 (CD54/ICAM1) and the vascular cell adhesion molecule (VCAM), which are typically expressed by the classical antigen-presenting cells, as well as the cell surface receptor CD40 and several toll-like receptors (TLRs) [24,29].

#### 2.1.1. CD40/CD40L

CD40 is a member of tumor necrosis factor (TNF) gene superfamily expressed in various cell types, including typical antigen-presenting cells, epithelial cells, and B cells [45]. CD40 binds to its ligand (CD40L/CD154), which is also expressed in SGECs, i.e., mainly the ductal cells, and infiltrating lymphocytes, and CD40 signaling induces the expression of ICAM1 on SGECs and the activation of T cells, further supporting the continuous interplay between SGECs and inflammatory cells in SjD pathogenesis [29,45]. Increased salivary levels of CD40L have been correlated with reduced salivary flow in SjD patients [46] and targeting the CD40/CD40L axis appears as a promising therapeutic strategy [45]. Treatment with an anti-CD154 antibody in a non-obese diabetic (NOD) mouse model of SjD resulted in significantly decreased presence of infiltrating T cells, B cells, and macrophages within the salivary glands, accompanied by reduced auto-antibody secretion [47]. Similarly, CD40 DNA vaccination in NOD mice resulted in decreased lymphocytic infiltration in the salivary gland and markedly increased salivary flow rate [48]. Recently, significant improvement of the unstimulated and stimulated salivary flow rates was observed in a randomized, double-blind, placebo-controlled, phase IIb clinical trial evaluating the safety and efficacy of an anti-CD40 monoclonal antibody (iscalimab) in SjD patients [49]. In another recent randomized, double-blinded, placebo-controlled, phase II clinical trial of a CD40L antagonist (dazodalibep), a numerically greater improvement of stimulated salivary flow was found in SjD patients treated with the CD40L antagonist compared with the placebo receiving group, but this difference was not statistically significant [50].

#### 2.1.2. TLRs

TLRs are pattern-recognition receptors that initiate immune responses by recognizing pathogen-associated molecular patterns (PAMPs) released from microbes, as well as danger-associated molecular patterns (DAMPs) derived from endogenous cellular or extracellular matrix components [51,52]. TLRs are predominantly expressed in SGECs, but also in plasmacytoid dendritic cells (pDCs), monocytes, and macrophages [53]. TLR signaling promotes the activation of interferon regulatory factors (IRFs), e.g., IRF5 and IRF7, the production of type I interferons (IFNα/β), the induction of the Nuclear factor-κB (NF-κB) cascade, and the stimulation of Ro52 antigen presentation through the MHC class I, which collectively enhances the immune responses in SjD [53,54]. The role of several TLRs in SjD pathogenesis and innate immune responses in SGECs has been thoroughly described previously [29], and preclinical studies indicated a direct association between TLR activation and salivary gland dysfunction [51,53]. Experiments in mouse models of SjD showed significantly reduced pilocarpine-stimulated salivary flow rate in mice treated with the TLR7 agonist imiquimod [55], the TLR3 agonist polyinosinic:polycytidylic acid (poly(I:C)) [56,57], and the TLR4 agonist lipopolysaccharides [58], whereas treatment with the TLR9 agonist BL-7040 or the TLR9 oligonucleotide inhibitor ODN2088 resulted in elevated saliva levels [59]. Moreover, although no significant difference in the pilocarpine-stimulated salivary flow rate was observed in NOD mice after TLR7 knockout [60], a significant increase in salivary flow rate was observed in TLR7 knockout mice after induction of the lysosome-associated membrane protein 3 (LAMP3) expression with an adeno-associated virus serotype 2 vector in another study [61]. In line with these preclinical findings, a systematic review and meta-analysis reported that the administration of the TLR7 inhibitor hydroxychloroquine in patients with primary SjD resulted in statistically significant improvement of oral dryness symptoms and significantly higher levels of unstimulated salivary flow rate [62]. In contrast, no significant differences in the unstimulated and stimulated salivary flow rate were noted in a randomized, double-blind, placebo-controlled, phase II clinical trial in the SjD patients receiving the RNase Fc fusion protein RSLV-132 that degrades TLR ligands compared with the placebo group [63].

### 2.2. IFN–JAK–STAT Axis

IFNs are a superfamily of cytokines classified in three classes, i.e., type I, II, and III, that participate in innate and adaptive immune responses, induced by exogenous (e.g., viral infections) or endogenous (e.g., retroelements) trigger factors [23]. IFNs primarily signal through the JAK—STAT pathway, inducing the overexpression of numerous IFN—related genes (“IFN signature”) identified in the minor salivary glands and the peripheral blood of SjD patients [64,65]. The JAK—STAT axis includes four JAK intracellular tyrosine kinases, i.e., JAK1, JAK2, JAK3, TYK2, and seven STAT transcription factors, i.e., STAT1, STAT2, STAT3, STAT4, STAT5a, STAT5b, and STAT6 [64].

#### 2.2.1. Type I IFNs

Type I IFNs (predominantly IFNα and IFNβ) are mainly expressed by pDCs, but also epithelial cells, monocytes, and fibroblasts, and are considered as major inflammatory drivers in SjD [65]. IFNα and IFNβ binding to the type I IFN receptor (IFNAR), composed of the IFNAR1 and IFNAR2 subunits, results in JAK1 and TYK2 activation, and subsequently to STAT1 and STAT2 phosphorylation and nuclear translocation, ending up in promoting inflammatory and antiviral responses [66]. A recent study based on weighted gene co-expression network analysis, coupled with machine learning, identified the “response to type I interferon signaling pathway” and “cellular response to type I interferon” as the top enriched biological processes among the differentially expressed genes in SjD [67]. Moreover, stimulation of SGECs with IFNα induced the upregulation of *LAMP3* [61]. *LAMP3* mRNA was elevated in the minor salivary glands of primary SjD patients compared with normal subjects, while *in vitro* experiments in salivary gland cell lines revealed that LAMP3 impairs the autophagic degradation of caspase-8 and results in caspase-8 accumulation and lysosomal membrane permeabilization, promoting the lysosome-dependent epithelial apoptosis and, thus, the salivary gland epithelial dysfunction in SjD [68]. Paradoxically, a statistically significant increase in the unstimulated salivary flow rate, while not in the stimulated salivary flow rate, was observed in low-dose INFα-medicated SjD patients compared with placebo controls, according to a study that reported outcomes from two randomized, double-blind, placebo-controlled phase III clinical trials [69]. Due to the established role of type I IFNs in SjD pathogenesis, ongoing therapies with monoclonal antibodies target the main cellular source of type I IFNs, i.e., the pDCs, as well as the IFNAR [65], while LAMP3-induced lysosomal dysfunction might also represent a future targeted therapy for SjD [61,70].

#### 2.2.2. Type II IFNs

Type II IFN, i.e., IFNγ, is also implicated in SjD pathogenesis [24]. IFNγ is highly expressed by activated natural-killer (NK) cells and T cells, but also by B cells, macrophages, DCs, and innate lymphoid cells [64,65]. IFNγ binds to a different receptor than IFNα and IFNβ, which is dimerized to IFNGR1 and IFNGR2 subunits, inducing JAK1 and JAK 2 activation, and next STAT1 homodimerization and nuclear translocation, resulting in the transcription of IFNγ-related genes [65]. In SjD, IFNγ enhances the antigen-presenting properties of SGECs via promoting the expression of MHC class II and costimulatory molecules on them and exerts a pro-inflammatory action via inducing the secretion of various cytokines and chemokines [44]. In addition, IFNγ impairs the salivary gland function in SjD patients by altering the expression levels of microRNAs in the minor salivary glands, e.g., hsa-miR-513c-3p and hsa-miR-424–5p, that regulate genes involved in signaling pathways associated with saliva secretion [71].

#### 2.2.3. Type III IFNs

Type III IFNs, i.e., IFNλs, have also been involved in SjD pathogenesis, as increased expression of IFNλs and their receptors was detected in the acinar and ductal cells of minor salivary glands in SjD patients compared with non-SjD sicca controls [72,73]. Similarly to type I IFNs, IFNλs signaling results in JAK1 and TYK2 activation, and STAT1 and STAT2 phosphorylation, nuclear translocation, and mediation of IFN-related gene expression [65].

#### 2.2.4. JAK—STAT Signaling

The JAK—STAT axis is one of the most enriched signaling pathways among differentially expressed genes in the minor salivary glands of SjD patients [74] and represents a promising therapeutic target of current treatment avenues, including the pan-JAK inhibitor (tofacitinib), the JAK1 selective inhibitor (filgotinib), the JAK1/JAK2 inhibitors (baricitinib, ruxolitinib), and the selective TYK2 inhibitor (deucravacitinib) [64,65]. JAK—STAT pathway inhibition in a mouse model of SjD via filgotinib resulted in significantly increased salivary flow rate [75]. However, no significant difference was observed in the unstimulated and stimulated salivary flow rates of patients with primary SjD or SjD associated with other autoimmune diseases after a 24-week placebo-controlled treatment period of filgotinib administration in a multicenter, randomized, double-blind, placebo-controlled, phase II clinical trial [76]. Moreover, treatment of SjD murine models with the non-selective JAK inhibitor tofacitinib resulted in reduced lymphocytic infiltration of the salivary glands and a significant increase in the salivary flow rate [77,78], while tofacitinib administration in salivary gland organoids from SjD patients enhanced their swelling capacity [79]. As the swelling assay is considered indicative of the saliva secretion capacity, these findings indicate that tofacitinib might promote salivary flow restoration [79]. Finally, promising results regarding a significant improvement in disease activity scores, including the EULAR Sjögren’s Syndrome Patient Reported Index (ESSDAI), measuring dryness, fatigue, and pain, were recently observed in a phase II clinical trial assessing the efficiency of tofacitinib in SjD patients [80]; however, salivary flow rate data were not specified.

### 2.3. Cytokine and Chemokine Amplification

In addition to IFNs, numerous other cytokines are implicated in the development and progression of SjD, including pro-inflammatory (e.g., interleukin (IL)-1b, IL-6, IL-8, IL-17, IL-23, IL-33, TNFα), anti-inflammatory (e.g., IL-4, IL-10), and pro-survival cytokines (e.g., the B-cell activating factor (BAFF)) and chemokines (e.g., C-X-C motif chemokine ligand (CXCL) 9, CXCL10, CXCL11, CCL3/MIP1a, and CXCR3) [27]. ILs are cytokines produced mainly by T cells; they induce recruitment of adaptive immune cells and promote salivary gland inflammation and atrophy of secretory cells, thus resulting in impaired salivary flow production and secretion [2,27]. Chemokines are leukocyte attractants produced by SGECs that promote inflammatory infiltration by recruiting immune cells to the salivary glands [81,82].

The expression of several cytokines has been correlated with the focus score in the salivary gland biopsy of SjD patients. In particular, the focus score exhibited a statistically significant positive correlation with the immunohistochemical expression of IL-25 (IL-17E) [83] and the mRNA expression of *IL-40* [84] and *CXCL10 (IP-10)* [85] in the minor salivary glands, as well as with the salivary levels of IL-1b and IL-4 [46] and with the serum levels of CXCL11 [81] and CXCL13 [86]. Moreover, clinical parameters of xerostomia have been related to cytokines’ expression in SjD patients. In particular, salivary flow of SjD individuals, measured as whole saliva or salivary flow rate, has been negatively correlated with the salivary levels of IL-1b [46,87] and IL-17 [88], whereas it has been positively associated with the salivary levels of IL-23 [46]. Moreover, a statistically significant negative association has been observed in SjD patients between the unstimulated salivary flow and the salivary levels of IL-6, IL-8, IL-10 [87], and CCL3/MIP1a [39]; between the stimulated salivary flow and the salivary levels of CCL3/MIP1a [39]; between the liquid (serum) fraction of the unstimulated salivary flow and the salivary levels of IL-6, IL-8, IL-10, IL-17A, and TNFα; and between the liquid (serum) fraction of the stimulated salivary flow and the salivary levels of IL-6 and IL-8 [87]. In contrast, no significant association was noted between the cytokines’ salivary levels and the foamy fraction of unstimulated or stimulated salivary flow [87], as well as between serum levels of CXCL9, CXCL10, CXCL11, and CXCR3 and the unstimulated or stimulated salivary flow rate [81]. The negative correlation between most cytokines and salivary flow might be explained by different nervous regulation as cytokine secretion is predominantly regulated by the sympathetic nervous system, while the liquid fraction of salivary flow is regulated by the parasympathetic nervous system that is disturbed in SjD patients [87].

In an experimental SjD mouse model, IL-33 signaling was associated with decreased salivary flow rate, while a significantly increased salivary flow rate was seen in IL-33 knockout mice [89]. In another experimental SjD mouse model, a significantly increased salivary flow rate was observed in mice treated with the anti-IL-17 neutralizing antibody [90]. However, systemic treatments targeting cytokines have not proved efficient in decreasing the disease severity of SjD patients [24]. Moreover, the double-blind randomized placebo-controlled clinical trials of the IL-6 receptor inhibitor tocilizumab [91], or the TNF-signaling inhibitors infliximab [92] and etanercept [93], resulted in no significant improvement in the salivary flow rate of SjD patients.

#### BAFF/BAFF Receptor (BAFFR)

BAFF, encoded by the TNFSF13B gene, is a pro-survival cytokine and member of the TNF superfamily that, binding to its receptor BAFFR, activates the phosphoinositide 3-kinase (PI3K)-protein kinase B (Akt)-mammalian target of rapamycin (mTOR) and NF-κB-signaling pathways, thus promoting the synthesis of pro-inflammatory molecules and inducing an intense inflammatory response in SjD patients [2,25]. BAFF plays a critical role in SjD pathogenesis, reflected in both systemic, i.e., in serum, and local, i.e., in salivary glands, effects, and acts as a bridge between the innate and adaptive immunity [94,95,96]. Serum BAFF enhances the survival and differentiation of B cells [95], and increased BAFF levels in serum of SjD patients significantly correlate with the ESSDAI score [97], as well as the levels of anti-SSA/Ro and anti-SSB/La antibodies, the rheumatoid factor levels, and with hypergammaglobulinemia [98]. BAFF expression in salivary glands is significantly increased in SjD patients and is mainly induced by type I and II IFN signaling. BAFF is secreted by the SGECs and innate immune cells, as well as T cells and B cells, and it promotes antibody production and the ectopic germinal center-like structure formation in SjD [95,96]. In SjD patients with ectopic germinal center-like structures in minor salivary gland biopsy, serum BAFF levels have been positively correlated with the focus score [99]. Moreover, in a NOD mouse model of SjD, the administration of a B-cell depleting, mouse BAFF-inhibiting monoclonal antibody resulted in a significant decrease in the focus score of lymphocytic infiltrates in the submandibular glands of mice and a significant increase in the stimulated salivary secretion [100].

Although several drug therapies targeting BAFF have shown some promising results in SjD [24,26], their efficacy in management of hyposalivation is still to be elucidated. Intravenous administration of the monoclonal antibody belimumab, which inhibits BAFF signaling, showed no significant change in the biopsy focus score and the unstimulated whole salivary flow levels of SjD patients [101]. When subcutaneous belimumab was combined with the intravenous anti-CD20 monoclonal antibody rituximab in SjD subjects, a higher unstimulated salivary flow (although not specified to be statistically significant), as well as a trend towards higher stimulated salivary flow, was observed [102]. Intravenous treatment with ianalumab, a monoclonal antibody (VAY736) against BAFFR that exerts a dual role of BAFFR pathway blockage and B cell depletion, led to a temporary increase in unstimulated and stimulated salivary flow that decreased again at the end of the study [103]. In contrast, a randomized, double-blind, placebo-controlled, phase-2 clinical trial of subcutaneous ianalumab in SjD patients resulted in significantly elevated levels of stimulated salivary flow [104]. Another randomized, double-blind, placebo-controlled, phase-2 clinical trial tested telitacicept, a fusion protein that inhibits both BAFF and the proliferation-inducing ligand (APRIL), in SjD patients, but no significant change in unstimulated salivary flow rate was found [105].

### 2.4. Organization of the Inflammatory Milieu in Salivary Glands

Innate sensing in SGECs of SjD patients induces the activation of IFN-JAK-STAT-signaling pathway and the amplification of numerous cytokines and chemokines that promote the adaptive immune cell responses governed by T cells and B cells [94,106]. RNA-sequencing-based transcriptomic profiling of paired parotid and minor salivary glands of SjD patients indicated strong enrichment of immune pathways associated with T cell and B cell activation, including CD3/CD28 T cell activation and CD40 signaling in B cells, as well as the IFNα- and TLR7-signaling pathways [107]. Using multiparameter flow cytometry, CD45^+^ leukocytes and CD3^+^ pan-T cells emerged as the dominant immune populations in SjD patients as they were derived from minor salivary glands, with CD8^+^ T cells and CD19^+^ B cells representing the major subsets within the infiltrates, whereas CD4^+^ T cells were present at comparatively lower frequencies [108]. A leukocyte subset not assigned to T cells or B cells was also identified, probably representing other immune cell populations, including DCs, monocytes/macrophages, and NK cells [108]. Another recent study employing single-cell RNA sequencing and multiplex immunofluorescence, complemented by spatial proteomics/transcriptomics of minor salivary glands of SjD patients, also identified CD45^+^ hematopoietic cell clusters comprising CD4+ and CD8+ CD3+ T cells, B cells, plasma cells, and myeloid cells [109].

#### 2.4.1. T Cells

T cells are attracted to the salivary glands of SjD patients, following the local secretion of IFN-induced cytokines and chemokines by the SGECs, and, next, activated T cells produce IFNγ and further interact with the SGECs via the IL-7/IFN axis [110].2.4.1.1. CD4+ T cells

CD4+ T cells, including T helper (h) 1, Th2, Th17, T follicular helper (Tfh), and regulatory T cells (Tregs) subsets, contribute to the organization of the inflammatory microenvironment in salivary glands of SjD patients [2]. Effector Th1 and Th17 cells produce pro-inflammatory cytokines such as IFN-γ, IL-17, and IL-22; Th2 secretes IL-4 and IL-5 to stimulate B cells, inducing antibody formation; Tfh cells promote B-cell differentiation and germinal center formation through IL-21 and CD40L signaling; and Tregs exert immunoregulatory effects via IL-10 and TGF-β [2,24,106,111].

##### CD8+ T Cells

CD8+ T cells also participate in SjD pathogenesis by driving salivary gland tissue injury [112]. Activated CD8+ T cells secrete pro-inflammatory molecules, e.g., IFNγ and TNFα, release cytotoxic mediators, including perforin and granzymes (e.g., GZMA, GZMB, GZMK), and activate the Fas surface death receptor-signaling pathway that collectively induces apoptosis of the SGECs [2,106]. Utilizing a quantitative multicolor immunofluorescence analysis, Kaneko et al. [112] identified CD8+ cytotoxic T cells as the prevailing inflammatory population in the minor salivary gland infiltrates of SjD patients and showed that GZMA+CD8+ cytotoxic T cells expressing Fas ligand (FasL) were in close proximity to Fas-expressing apoptotic epithelial cells, which mainly comprised acinar and ductal cells. Another study using single-cell and spatial transcriptomics coupled with spatial immunophenotyping on minor salivary glands of SjD patients highlighted a SjD-enriched GZMK+CD8+ T cell subpopulation spatially associated with SGECs characterized by an acinar differentiation gene profile, whose abundance positively correlated with biopsy focus score [113]. The latter finding was in line with an immunohistochemical study reporting that CD8+ T cell counts in minor salivary glands were significantly higher in SjD patients with a focus score ≥1 than those with a focus score <1 or health controls [114]. Moreover, a recent study employing machine learning of digitalized slides of minor salivary gland tissues derived from SjD and non-SjD sicca controls identified the presence of CD8+ T cells spatially associated with seromucous acinar cells as a SjD characteristic histopathological feature [115].

##### MAIT Cells

Moreover, a subset of innate-like T cells, mainly found in mucosal tissues, termed mucosal-associated invariant T (MAIT) cells, has been linked to SjD pathogenesis. MAIT cell counts were significantly lower in the peripheral blood of SjD patients despite their increased secretion of pro-inflammatory cytokines (e.g., IFNγ and TNFα) [116,117]. In contrast, MAIT cells were identified in the minor salivary glands of SjD patients, but not in those of healthy controls [116]. The reduced frequency of MAIT cells in peripheral blood, together with their presence in inflamed salivary glands, could suggest their increased cell death or their redistribution toward target tissues, where they may contribute to local immune activation and epithelial injury [116]. Thus, the precise role of MAIT cells in SjD warrants further investigation.

##### Targeting T Cells

As T cells express on their cellular surface CD28 that binds the stimulatory CD80/CD86 or the inhibitory cytotoxic T-lymphocyte-associated protein 4 (CTLA4) on the cellular surface of antigen-presenting cells [118], the CTLA4-Immunoglobulin (Ig) G fusion protein abatacept that inhibits the interaction between CD80/CD86 and CD28 has emerged as a potential therapeutic avenue in SjD [24,26]. One small, open-label trial showed some promising results with intravenous abatacept, contributing to an increased unstimulated salivary flow rate [119], in contrast to a previous open-label study that had not reported a statistically significant improvement in unstimulated or stimulated salivary flow rate following treatment with intravenous abatacept [120]. However, two randomized, double-blind, placebo-controlled, phase III clinical trials did not result in statistically significant improvement of unstimulated or stimulated whole salivary flow with subcutaneous abatacept injections [121,122], while temporary, significant elevation in unstimulated and stimulated whole salivary flow levels was observed during subcutaneous abatacept treatment in another randomized clinical trial that started as double-blind, placebo-controlled until the 24th week and continued as an open-label trial until the 48th week [123]. Moreover, abatacept therapy did not result in significant changes in the biopsy focus score of parotid and minor salivary glands of SjD patients [124,125].

Activated T-cells express the inducible T-cell co-stimulator (ICOS) protein, which has been found to be upregulated in the salivary glands, saliva, and whole blood of SjD patients [126], while *ICOS* mRNA expression in salivary glands of SjD patients significantly correlated with the biopsy focus score [127]. Moreover, repeated salivary gland biopsies prior and after treatment with the anti-ICOS ligand monoclonal antibody prezalumab revealed a significantly lower number of infiltrating CD4+/ICOS+ Tfh-like cells in salivary glands, indicating a potential glandular effect of this treatment that merits further evaluation [128]. In addition, a double-blind, placebo-controlled randomized clinical trial assessed the efficacy of low-dose IL-2 in patients with primary SjD, as IL-2 at low levels suppresses Tfh and Th17 cells differentiation and contributes to a selective increase in Tregs [129]. The treatment effect on salivary glands was investigated via ultrasonography, and no significant changes were found, while salivary flow rate was not evaluated [129].

New therapeutic avenues of chimeric antigen receptor-T cell treatment are under investigation [106] and have shown promising results regarding the alleviation of salivary gland inflammatory infiltrates in experimental mouse models [130]. Encouraging results have also emerged from laboratory studies that evaluated the efficacy of fingolimod (FTY720), an antagonist of sphingosine-1-P (S1P), in SjD mouse models [131,132]. S1P receptor 1 is expressed on T cells and B cells and, by binding to its ligand S1P, promotes the lymphocyte migration from secondary lymphoid organs to the target organs; thus, the S1P antagonist inhibits this migration and has been shown to improve the salivary flow rate in SjD mice [131,132]. Finally, future perspectives exist regarding mesenchymal stem cell (MSC)-based therapies in SjD patients, as MSC treatment suppresses Th1, Th17, and Tfh cells and induces Tregs, and, thus, attenuates the autoimmune responses [31]. Administration of umbilical cord MSCs in 24 SjD patients with dry mouth has resulted in a significant increase in unstimulated and stimulated salivary flow rate [133], while a significant improvement in salivary flow rate was also observed in an experimental mouse model of SjD after treatment with labial salivary gland-derived MSCs [134].

#### 2.4.2. B Cells

B cells play an essential role in SjD pathogenesis by producing autoantibodies, such as antinuclear antibodies, rheumatoid factor, anti-SSA/Ro, and anti-SSB/La antibodies, that target the exocrine glands, resulting in inflammatory infiltration and glandular destruction [2,111]. The reciprocal interactions between B cells and SGECs in SjD contribute to disease progression, as the SGECs secrete various proinflammatory cytokines and chemokines, e.g., BAFF, CXCL10, and CXCL13, that induce the survival and activation of B cells, while the activated B cells infiltrate the salivary gland epithelium, promoting the formation of lymphoepithelial lesions and the apoptosis of the SGECs [27,30,135,136].

##### B Cells Subpopulations

In addition to naïve B cells, several other B cell subpopulations have been identified in the salivary glands of SjD patients [2,28,111,137]. Among them are transitional B cells that had recently migrated from the bone marrow to secondary lymphoid organs and CD27+ memory B cells that are reduced in the peripheral blood of SjD patients, while they accumulate within the salivary glands, overexpress the CXCR4 and CXCR5 that mediate their response to SGECs-derived CXCL12 and CXCL13, and induce the formation of ectopic GC-like structures [137,138,139,140,141]. CD138+ plasma cells, differentiated from plasmablasts, produce autoantibodies and promote further inflammatory infiltration, while their increased expression in SjD salivary glands correlates with autoantibody positivity, serum IgG levels, and disease activity [142,143,144]. CD11c+ age-associated B cells (ABCs) exhibit increased expression of T-box transcription factor 21 that induces IFN-g and IL-12, thus promoting Th1 cell-mediated immune responses [145,146]. A recent study using a SjD mouse model showed that ABC expression in salivary glands increased with age and was positively correlated with Tfh cell expression [147]. Fc receptor-like 4 (FcRL4)+ B cells are observed near or within the ducts of inflammatory salivary glands of SjD patients and express CCR5, as well as CXCR3 and CD11c and CD97 adhesion molecules that facilitate cell-immune interaction with ductal SGECs-expressing CXCL10 and BAFF [148,149,150]. FcRL4+ B cells are more frequent in the parotid gland than in labial salivary glands, and they exhibit significantly higher mRNA expression in primary SjD-related parotid MALT lymphomas compared with parotids of non-lymphoma SjD patients, thus representing a potential source of progenitor cells for MALT lymphoma development in primary SjD patients [148]. Marginal zone (MZB) B cells are mainly found in the marginal region of spleen, while they accumulate within the salivary glands of SjD patients, promoting the antibody-mediated destruction of the glandular tissue [151], while suppression of MZB cells in a SjD mouse model resulted in absence of autoantibodies, normal salivary gland histology, and restoration of physiological saliva secretion [152]. Moreover, MZB cells comprise a major cell source for non-Hodgkin lymphoma development [153]. Regulatory B cells (Bregs) secrete IL-10, IL-35, and GZMK, hinder Th1 and Th17 responses, and induce Tregs expression, thus promoting an anti-inflammatory immune reaction, and their expression decreases in advanced phases of SjD [111,154,155]. IgD- CD27- double negative B cells are detected in lower levels in blood of SjD individuals than in healthy controls, and their expression correlates significantly with glandular involvement, disease activity, and higher risk for lymphoma development [94,156].

##### B Cells Activation

B cells stimulation is triggered by activated T cells through the production of pro-inflammatory cytokines, while BAFF is a key regulator of B cell proliferation, maturation, and survival [2]. B cells express transmembrane antigen receptors, i.e., B-cell receptors (BCR), that are fundamental for the B cell response to pathogens, antibody production, and maintenance of B cell survival. Antigen engagement initiates BCR signaling, involving kinases, e.g., the spleen tyrosine kinase (SYK) that phosphorylates downstream targets and activates the Bruton’s tyrosine kinase (BTK), as well as enzymes, e.g., PI3Kδ [95]. SYK is a cytoplasmic tyrosine kinase, mainly expressed in B cells, T cells, DCs, and NK cells, and SYK signaling induces the production of pro-inflammatory cytokines [157,158]. Similarly, BTK, expressed by B cells and myeloid cells, promotes antibody production and the stimulation of pro-inflammatory cytokines [159]. PI3Kδ is an upstream activator of PI3K-Akt-mTOR pathway, is predominantly expressed in leukocytes, and is significantly involved in cell growth, survival, and differentiation [160]. *PIK3CD* mRNA, which encodes PI3Kδ, was expressed in the salivary gland of primary SjD patients and significantly correlated with the biopsy focus score [161]. Moreover, phosphorylated ribosomal protein S6 (pS6), i.e., a downstream molecule of PI3K-Akt-mTOR pathway, was also expressed in SjD salivary glands and colocalized with CD138+ plasma cells [161]. Hyperactivity of B cells in primary SjD is also mediated by mTOR complex 1 (mTORC1)—glucose transporter 1 (GLUT1) glycolysis pathways, and primary SjD patients-derived B cells appeared more metabolically active and exhibited higher glycolysis capacity than those from non-SjD controls [162]. *In vitro* treatment of B cells from primary SjD patients with 2-deoxy-D-glucose or metformin inhibits glycolysis-suppressed B cell activation, indicating that mTORC1-GLUT1-signaling inhibition might be a potential new therapy for SjD [162]. In addition, B-cell hyperactivity in primary SjD is driven by the Ribonucleotide Reductase Regulatory Subunit M2 (RRM2) through modification of N6-methyladenosine (m6A), i.e., an RNA modification that acts as a crucial post-transcriptional regulator of gene expression [163]. Increased RRM2 expression was observed in lymphocytic infiltrates and periductal regions in salivary glands of primary SjD patients, and RRM2 levels showed a significantly negative correlation with salivary flow rate of primary SjD individuals [163]. Thus, RRM2 represents a promising therapeutic target of SjD that should be further evaluated [163].

##### Targeting B Cells

B-cell hyperactivity is a pathogenetic hallmark of SjD; thus, B-cell targeting emerges as a dominant therapeutic approach [26]. In addition to drugs targeting BAFFR, IFNs via TYK, and the CD40-CD40L pathway, which indirectly reduce B-cell survival and have been already described in previous sections, B-cell targeting in SjD patients includes therapies inducing B-cell depletion and inhibitors of B-cell surface marker CD22, as well as targeting of BCR signaling and B cells via modulation of antibody presentation [24,26]. B-cell depletion of SjD patients with the anti-CD20 monoclonal antibody rituximab resulted in statistically significant improvement of unstimulated [164] or both unstimulated and stimulated salivary flow [165] in two randomized, placebo-controlled clinical trials, whereas no significant difference in salivary flow was found in two other studies [166,167]. Unstimulated whole salivary flow improvement by at least 20% was observed in 7 out of 15 patients receiving anti-CD22 monoclonal antibody epratuzumab in an open-label, phase I/II study [168], while information regarding any salivary flow changes among 13 SjD patients with clinical response to anti-CD20 monoclonal antibody obinutuzumab was not available [169]. Moreover, no significant changes in salivary flow rate of SjD patients were found after BCR signaling targeting with the SYK inhibitor lanraplenib [76], the BTK inhibitors tirabrutinib [76] and remibrutinib (despite a trend towards salivary flow improvement) [170], the selective PI3Kδ inhibitor seletalisib [171], or after targeting B cells via the cathepsin S inhibitor-mediated MHC class II–restricted antigen presentation [172] or via after therapy with the lymphotoxin β receptor IgG fusion protein (LTβR-Ig) baminercept that inhibits the LTβR-signaling pathway that is required for homing of B cells and T cells into secondary lymphoid organs and inflamed tissues [173].

### 2.5. Salivary Epithelial Tissue Destruction and Secretory Dysfunction

The orchestration of an inflammatory milieu within the salivary glands and the chronic exposure of SGECs to pro-inflammatory signals promote the initiation of epithelial stress responses that progressively disrupt the structural integrity, metabolic regulation, and secretory competence of salivary gland parenchyma [2,174]. These responses include programmed cell death pathways, mitochondrial dysfunction, structural changes in both acinar and ductal cells, e.g., acinar atrophy, duct dilatation, and narrowing, and formation of lymphoepithelial lesions that comprise hyperplastic ducts and lymphocytic infiltrates, as well as loss of junctional integrity and quantitative and qualitative aberrations in the salivary secretory process [2,30,174].

#### 2.5.1. Cell Death Mechanisms in SGECs

Innate sensing of SGECs and excessive inflammatory response in the salivary glands of SjD patients induce the death of SGECs via various modes, including apoptosis, autophagy, pyroptosis, and ferroptosis, which, in turn, triggers the release of autoantigens from the dead SGECs and results in the amplification of immune responses and the glandular destruction and dysfunction [29,175].

##### Apoptosis

Apoptosis is a programmed cell death of SGECs in SjD, mediated through coordinated extrinsic and intrinsic pathways that end up in caspase cascade activation, while apoptotic signals are promoted via the NF-κB and STAT3–IκBζ [175] signaling pathways and several apoptosis-related cytokines, including LAMP3 [176]. The extrinsic pathway initiates via binding of the FasL and TNF-related apoptosis-inducing ligand (TRAIL) on T cells with the Fas and death receptor 5, respectively, on SGECs, and results in caspase 8/3 cleavage [112,175,177]. TRAIL also induces the intrinsic apoptotic pathway that triggers the mitochondrial release of cytochrome c, as well as formation of apoptosome and caspase 9/3 cleavage [175,177].

##### Autophagy

Autophagy is a protective cellular process, activated in response to stress conditions, in which intracellular proteins and damaged organelles are sequestered into autophagosomes that are submitted to lysosomal degradation [175]. Upregulation of autophagy pathways has been observed in SjD patient-derived SGECs compared with sicca controls, while the expression of the autophagy-related protein LC3-IIB was shown to positively correlate with the expression of adhesion molecules (e.g., ICAM) in activated SGECs [178].

##### Pyroptosis

Pyroptosis is an inflammatory type of programmed cell death, triggered by endogenous danger signals or intracellular pathogens [179]. It is induced by the activation of a multiprotein intracellular complex, termed the inflammasome, resulting in changes in cell osmotic pressure, cell swelling, and, finally, cell membrane rupture [179]. Recent studies integrating bioinformatics analyses identified a pyroptosis-related gene signature in the peripheral blood [180] and in salivary glands [181] of SjD patients. Special focus has been attributed to the P2X7 receptor (P2X7R)—NOD-like receptor P3 (NLRP3) inflammasome complex in SjD that, upon activation, induces the release of the pro-inflammatory cytokines IL-1β and IL-18 [182]. A significantly higher expression of P2X7R, as well as of the inflammasome components NLRP3 and caspase-1, was identified in the salivary glands of SjD patients compared with non-SjD controls, accompanied by significantly elevated IL-18 protein levels in SjD patient saliva [182]. Moreover, the mRNA of gene coding for the inflammasome components caspase-1 (*CASP1*) and Gasdermin D (*GSDMD*) was overexpressed in the SGECs of SjD patients and was significantly correlated with type I IFN signature genes, while type I IFN treatment in human SGECs induced caspase-1 and GSDMD activation, indicating the promoting role of type I IFN signaling for inflammasome-related pyroptosis in SjD [183]. 

##### Ferroptosis

Ferroptosis is a form of programmed cell death, caused by iron overload and impairment of the glutathione—glutathione peroxidase 4 (GPX4) axis-mediated antioxidant defenses, resulting in accumulation of reactive oxygen species (ROS) and lipid peroxides, and rupture of the cell membrane [184]. Current evidence suggests that IFNγ signaling via the JAK/STAT1 axis induces ferroptosis in SGECs in SjD, resulting in impaired saliva secretion [185,186]. Galectin 9 (Gal9) also emerged as a potential upstream regulator of ferroptosis via induction of IFNγ and downregulation of GPX4 in SjD, and serum Gal9 levels showed a strong positive and negative correlation with focus score and unstimulated salivary flow, respectively, of SjD patients [187]. GPX4 knockdown promoted phosphorylation and nuclear translocation of STAT4 in SGECs, facilitating binding of pSTAT4 within the promoter of water channel protein aquaporin 5 (AQP5), thus suppressing AQP5 expression [188]. Notably, pharmacological inhibition of ferroptosis using apigenin [189], genistein [190], and melatonin [191] in mouse models of SjD resulted in improved salivary gland function, indicating that ferroptosis targeting might be of therapeutic interest in SjD and warrants further investigation.

#### 2.5.2. Mitochondrial Dysfunction in Salivary Glands

Mitochondria exert a crucial role in cellular homeostasis via adenosine triphosphate production and ROS regulation, and mitochondrial dysfunction contributes to increased oxidative stress and release of mitochondrial components that could act as DAMPs and induce prominent inflammatory response and epithelial damage, thus promoting SjD pathogenesis [192,193].

Studies using transmission electron microscopy have shown swollen mitochondria exhibiting less and abnormal cristae, and cytoplasmic lipid droplets in SGECs of SjD patients [194,195]. Transcriptomics analysis-based studies on labial salivary glands of SjD patients revealed significant upregulation of mitochondria-related genes (*CD38*, *CMPK2*, *TBC1D9*) that promote immune responses [195,196], as well as metabolic reprogramming of mitochondria in the SjD immune milieu, with distinct enriched mitochondrial pathways in innate and adaptive immune cells [196]. RNA sequencing has also shown significant enrichment of the interferon-stimulated gene (STING) pathway in SjD salivary glands [197]. Zong et al. [198] demonstrated that STING activation in SjD is driven by mitochondrial DNA release resulting from lactate-induced mitochondrial leakage in SGECs. Lactate is a widely studied terminal byproduct of glucose metabolism, and lactate accumulation in the infiltrated T cells can amplify inflammation by triggering metabolic alterations within immune cells [198]. Utilizing Ultra-high Performance Liquid Chromatograph Coupled Mass Spectrometry, Piacenza Florezi et al. [199] revealed an increased concentration of metabolites involved in oxidative stress, including lactate, as well as amino acids related to T cell proliferation in the saliva of SjD patients compared with healthy controls.

#### 2.5.3. Structural Changes and Junctional Integrity Loss in SGECs

Structural changes of salivary glands in SjD include alterations in the apical membrane, lateral membrane, and basement membrane of SGECs, as well as extracellular matrix remodeling [174]. The acinar cells of SjD salivary glands exhibit fewer microvilli and elevated expression of ezrin and phosphorylated ezrin proteins that, similar to AQP5, mislocate from apical to the basolateral region of the acini, probably contributing to the disruption of microvilli structure [200,201]. The tight junctions on the lateral membrane of SGECs are also affected in SjD and show reduced integrity of occludins, ZO-1, and claudin-1, -3, and -4 [202]. In a mouse model of SjD, enlargement of acinar tight junction width in submandibular glands was observed, resulting in epithelial barrier dysfunction [203]. *In vitro* experiments in the same study revealed that IL-17 derived from infiltrating lymphocytes mediated claudin-4 and ZO-1 downregulation through NF-κB signaling, leading to impaired tight junction integrity [203]. Recently, decreased expression of another tight junction protein, i.e., tricellulin, was found in the salivary glands of SjD patients and in the SjD mouse model that coincided with hyposecretion [204]. Downregulation of tricellulin was induced by IFN-γ via the JAK/STAT1/miR-145 axis, while tight-junction sealing with AT1001 or miR-145 inhibition restored the epithelial barrier integrity and alleviated hyposalivation, indicating that tricellulin might represent a future therapeutic target for SjD-related xerostomia [204]. As reviewed by Hou et al. [174], altered laminin expression and nidogen hydrolysis induce remodeling of the basal membrane, while activation of matrix metalloproteinases mediates degradation of basal membrane and extracellular matrix components, promoting detachment of SGECs and basal lamina, and facilitating the inflammatory infiltration of the glandular epithelium in SjD. Horeth et al. [205] revealed enrichment of immune cell function-related genes in acinar and ductal cells of the submandibular glands in NOD mice with primary SjD using bulk-RNA-sequencing and single-cell RNA sequencing analysis, and they proposed that the SGECs in SjD might change to a “hybrid epithelial/immune cell-like state” characterized by a gene expression profile reminiscent of immune cells. Moreover, a recent study showed a significantly increased dilation of striated ducts in inflamed salivary glands of SjD patients and SjD NOD.B10 mice, and overexpression of Th2-derived IL-4 in the dilated ducts [206]. IL-4 in SjD NOD.B10 mice activated the Sonic Hedgehog-signaling cascade that resulted in the upregulation of the mesenchymal marker *SNAI2* and the downregulation of the epithelial cell adhesion marker *CDH1*, thus compromising the ductal cell adhesion and promoting ductal dilation [206]. Interestingly, treatment with an IL-4 neutralizing antibody suppressed the ductal dilation and induced a significant increase in the salivary flow in NOD.B10 mice, indicating that IL-4 inhibition might be of therapeutic benefit in SjD [206].

#### 2.5.4. Quantitative and Qualitative Aberrations in Salivary Secretion

Salivary secretion is a calcium-dependent process that requires activation of muscarinic receptors, production of calcium waves, chloride secretion through calcium-activated chloride receptor Anoctamin 1 (TMEM16A), and AQP5-mediated water secretion [207]. Hyposalivation in SjD is a multifactorial phenomenon resulting from activation and dysfunction of M3 muscarinic ACh receptor (M3R), which fails to produce normal calcium waves, as well as from reduction of the inositol trisphosphate receptors (IP3R) 2 and 3 in the endoplasmic reticulum of acinar cells, which are critical for intracellular release of calcium [174]. Autoantibodies against M3R are frequently detected in SjD patients and have been shown to suppress normal M3R-mediated signaling and intracellular trafficking of the AQP5, resulting in dysregulated calcium signaling and impaired saliva secretion [208,209,210]. In addition, experiments in a mouse model of SjD revealed Th1 and Th17 reactivity against M3R, which contributes to glandular inflammation, and further M3R dysfunction [211]. The reduced expression and mislocalization of IP3R2 and IP3R3 observed in the acinar cells of minor salivary gland from SjD patients [212], together with increased spatial separation between IP3Rs and TMEM16A that has been recently shown in a mouse model of early SjD [212], diminish local calcium exposure, impairing saliva secretion. In contrast, increased mRNA expression of *ITPR2* coding for IP3R2 and *ITPR3* coding for IP3R3, as well as of Stromal Interaction Molecule 1 (STIM1) and Transient Receptor Potential Canonical 1 (TRPC1), was found in the minor salivary gland from SjD patients compared with sicca controls in another recent study [213], whereas decreased protein expression of STIM1 and STIM2 in the peripheral blood mononuclear cells and lymphocytic infiltrates within the submandibular glands of SjD patients compared with healthy controls had been reported previously [214]. STIM1 and STIM2 are endoplasmic reticulum calcium sensors that, together with the calcium-selective channel proteins Orai (Orai1, Orai2, and Orai3), are critical regulators of calcium signaling that is further modulated by STIM and Orai interactions with TRP channels, e.g., TRPC1 [215]. Interestingly, the T cell-specific [214] or Treg-specific [216] deletion of both STIM1 and STIM2 in mice resulted in SjD-like lymphocytic infiltration of the submandibular glands and reduced saliva secretion.

Qualitative alterations in saliva of SjD patients might also occur due to hyposulfation of major salivary mucins, e.g., MUC5B and MUC7, that impair the viscoelastic, hydrating, and lubricating properties of saliva [217,218]. Finally, ectopic localization of MUC5B and MUC7 in the extracellular matrix of SjD salivary glands can activate TLR4 in SGECs and trigger pro-inflammatory responses that exacerbate glandular dysfunction [219].

**Table 1:** Effect of targeted treatments on the unstimulated and stimulated salivary flow levels of SjD patients.

Evaluated Drug	Drug Target	Type of Study	Outcome on USFR/SSFR
Iscalimab	Anti-CD40 mAb	RDBPC, phase IIb	↑ USFR/SSFR [49]
Dazodalibep	CD40 ligand antagonist	RDBPC, phase II	nsd SSFR [50]
RSLV-132	RNase Fc fusion protein degrading TLR ligands	RDBPC, phase II	nsd USFR/SSFR [63]
IFNα	IFNα agonist	RDBPC (combined phase III results from two studies)	↑ USFR/nsd SSFR [69]
Filgotinib	JAK1 inhibitor	RDBPC, phase II	nsd USFR/SSFR [76]
Tocilizumab	Anti-IL-6R mAb	RDBPC	nsd USFR [91]
Infliximab	TNF-signaling inhibitor	RDBPC	nsd SFR [92]
Etanercept	TNF-signaling inhibitor	RDBPC	nsd USFR/SSFR [93]
Belimumab	BAFF-signaling inhibitor	Open-label clinical study	nsd USFR [101]
Belimumab + Rituximab	BAFF-signaling inhibitor + B cell depletion	RDB, phase II	↑* USFR/SSFR [102]
Ianalumab	Anti-BAFFR mAb (dual BAFF-signaling inhibitor and B cell depletion)	RDBPC, phase II	(↑) USFR/SSFR [103]
		RDBPC, phase II	↑ SSFR [104]
Telitacicept	BAFF/APRIL inhibitor	RDBPC, phase II	nsd USFR [105]
Abatacept	CD80/CD86 and CD28 interaction inhibitor	Open-label clinical study	↑ USFR [119]
		RDBPC, phase III	nsd USFR/SSFR [121]
		RDBPC, phase III	nsd USFR/SSFR [122]
		Open-label clinical study	(↑) USFR/SSFR [123]
		Open-label clinical study	nsd USFR/↓ SSFR [120]
Rituximab	Anti-CD20 mAb (B-cell depletion)	RDBPC	↑ USFR [164]
		RDBPC	↑ USFR/SSFR [165]
		RDBPC	nsd SFR [166]
		RDBPC	nsd USFR [167]
Epratuzumab	Anti-CD22 mAb	Open-label clinical study	↑* USFR [168]
Lanraplenib	SYK inhibitor	RDBPC, phase II	nsd SFR [76]
Tirabrutinib	BTK inhibitor	RDBPC, phase II	nsd SFR [76]
Remibrutinib	BTK inhibitor	RDBPC, phase II	nsd SFR [170]
Seletalisib	PI3Kδ inhibitor	RDBPC, phase II	nsd SFR [171]
RO5459072	Cathepsin S inhibitor	RDBPC, phase II	nsd SFR [172]
Baminercept	LTβR-Ig fusion protein inducing reduction of circulating T/B cells	RDBPC, phase II	nsd USFR/SSFR [173]

↑, statistically significant increase; ↑* increase, statistical significance not specified; (↑), temporary increase; ↓, statistically significant decrease; mAb, monoclonal antibody; nsd, not statistically significant difference; RDB, randomized, double-blind; RDBPC, randomized, double-blind, placebo-controlled; SSFR, stimulated salivary flow rate; USFR, unstimulated salivary flow rate.

## 3. Dental Caries

Caries formation is the result of the breakdown of the teeth’s hard tissues by acidogenic bacteria, i.e., *Lactobacillus acidophilus* and *Streptococcus mutans*, organized in the form of biofilm on the tooth surface [220]. Despite good oral hygiene, primary SjD patients harbor higher numbers of acidophilic and cariogenic bacteria, such as *Streptococcus mutans* and *Lactobacillus* species, than healthy controls [221]. A recent study utilizing 16S ribosomal DNA high-throughput sequencing, coupled by bioinformatics, analyzed dental plaque and saliva from primary SjD patients, and saliva from healthy donors [222]. This study indicated significant enrichment of *Prevotella* and *Veillonella* in the saliva and of *Fusobacterium*, *Actinomyces*, *Corynebacterium*, and *Leptotrichia* in plaque samples of SjD patients [222]. Caries is more prevalent and aggressive in primary SjD individuals [34]. It usually involves structurally compromised regions characterized by enamel and dentin loss, such as the tooth root and the cervical part of tooth crown, as well as on atypical surfaces, such as the lingual surface and the incisal edge and cusp of teeth [33,223]. Greater caries prevalence in SjD patients indicates an elevated risk for long-term tooth loss [224], an increased number of teeth restorations, and the need for more frequent dental visits [225,226].

Patients with primary SjD exert greater caries risk than patients with salivary dysfunction due to other factors [226,227,228,229], which has been mostly attributed to hyposalivation, i.e., reduced salivary flow associated with decreased secretion of immunoglobulin A, an antibody responsible for the prevention of dental caries [34]. Loss of mechanical cleaning through flushing of sufficient saliva results in residual food debris, trapped on the vestibular surfaces of the teeth, and, as a result, acidogenic bacteria accumulate rapidly and thrive in this poorly lubricated environment [227]. A second possible mechanism of aggressive carious activity in patients with primary SjD is the impaired buffering capacity of their saliva [229]. The low buffer capacity is directly related to reduced bicarbonate and phosphate concentrations, which are the main buffering agents in saliva. The decreased pH and buffering capacity create an acidic oral environment, leading to rapid development of caries, particularly on smooth surfaces. In addition, lower pH and buffer capacity imply inability of saliva to neutralize acids produced by cariogenic bacteria, leading to a prolonged acidic environment, favoring enamel erosion [227,228,229].

Another proposed mechanism implies that systemic inflammatory processes in primary SjD, orchestrated by different cytokines, may independently contribute to tooth destruction beyond reduced salivary flow rate and buffer capacity [87]. In a prospective cross-sectional clinical study, including 20 females, significant correlations between decayed teeth and three of the salivary cytokines analyzed, i.e., IL-6, IL-8, and IL-10, were observed [87]. These findings are in line with a previous study reporting a significant correlation between another salivary cytokine, i.e., IL-1b and the cariogenic *Streptococcus mutans* [230]. Finally, reduced MUC7 sialylation in SjD patients [218] may also contribute to their increased caries risk, as reduced MUC7 is considered a saliva risk factor for caries among adults [231].

## 4. Periodontitis

Periodontitis is a biofilm-mediated, irreversible chronic inflammation of the supporting tissues of tooth and the main reason for tooth loss in adults [232]. Although periodontitis has been linked to various systemic conditions, including autoimmune diseases, e.g., systemic lupus erythematosus and rheumatoid arthritis [233], most systematics reviews with or without meta-analysis failed to provide strong evidence supporting the association between periodontitis and primary SjD [224,234,235,236]. One systematic review and meta-analysis showed that the periodontal condition of patients with SjD was worse compared with the control group but emphasizes that no robust conclusions can be drawn due to the great heterogeneity of the included data [237].

The bacterial profile of primary SjD patients does not resemble the anaerobic microbial profile of periodontitis patients, as a higher prevalence of caries-associated bacteria *Streptococcus* spp., *Lactobacillus* spp., and *Viellonella* spp. is found in primary SjD patients [238]. However, higher oral microbial counts of *Prevotella* spp., a characteristic species belonging to the prevalent red complex of periodontitis [239], has been identified in primary SjD patients [238].

Aiming to identify a pathogenetic link between SjD and periodontitis, Scardina et al. [240] studied the vascular changes of the gingival tissues in primary SjD patients and found that patients with primary SjD exhibited specific patterns in gingival microcirculation, such as a “cobweb” configuration with reduced capillary caliber and increased tortuosity, suggesting a decrease in gingival blood supply and indicating a more inflamed state, susceptible to periodontitis. Periodontitis and SjD also share immune-inflammatory responses, mediated by the upregulation of common pro-inflammatory cytokines, such as IL-6 and IL-17 [235]. SjD patients with periodontitis exhibited higher serum and salivary BAFF levels than non-SjD controls with periodontitis [241], while primary SjD patients had higher salivary IP-10 and MIP-1α levels than healthy controls [39]. Interestingly, a recent study using single-cell transcriptomics analysis revealed a significant downregulation of the CD8+ naïve T cells proportion in both periodontitis and primary SjD, probably induced through activation of BACE2-CDKN1A-signaling pathway in both diseases [242].

## 5. Oral Candidiasis

Oral candidiasis is a fungal infection caused by overgrowth of *Candida* yeast, predominantly *Candida albicans*. Although *Candida albicans* is a commensal in healthy subjects, it can transit to an opportunistic pathogen under certain dysbiotic conditions that favor infection [243]. Oral candidiasis is a potential oral complication in primary SjD patients [244], manifested as reddish tongue with complete loss of lingual papillae, angular cheilitis, or denture stomatitis [6,35,226]. Hyposalivation of primary SjD patients is the principal factor that may lead to oral candidiasis, and *Candida* infection counts showed a significant negative correlation with unstimulated whole saliva and stimulated whole saliva of SjD patients [35,245]. The reduction in both unstimulated and stimulated salivary flow rates promotes a “dry mouth” environment that lacks the mechanical flushing action needed to clear *Candida* cells from the oral cavity [35,244]. SjD patients demonstrate an impaired salivary composition with a deficiency in histatins, antifungal peptides, lysozymes, and secretory IgA. In addition, the reduced buffer capacity creates an acidic environment, favoring *Candida* overgrowth [246]. Chen et al. [39] applied immunoassay technology to investigate cytokine concentration in saliva and tears of SjD patients and reported that the increased *Candida* score correlated with high saliva levels of IP-10, a chemokine produced in response to IFNγ [247].

## 6. Conclusions

**Figure 1:**
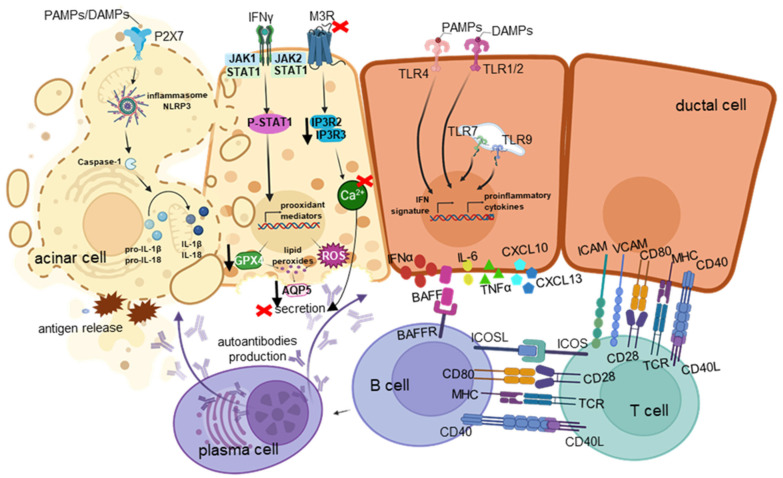
Core molecular mechanisms driving hyposalivation in SjD. Created with BioRender. Kalogirou, E. (2026) https://BioRender.com/252wxya, accessed on 27 April 2026.

The molecular basis of the oral manifestations of SjD has mainly resulted from the understanding of the pathogenetic mechanisms that govern the reduction of saliva secretion, leading to the appearance of xerostomia. Among these mechanisms (Figure 1), the immune sensing of the salivary gland epithelium and the activation of the IFN-JAK-STAT pathway play a dominant role, leading to a cytokine and chemokine cascade that promotes the coordinated recruitment of inflammatory cells, mainly of the T and B lymphocyte lineage. At an advanced disease stage, the destruction of the glandular epithelium, which in turn stimulates a further immune response, results in impaired secretory function and clinical manifestation of xerostomia, which predisposes to dental caries and candidiasis.

As summarized in Table 1, the impact of several new therapeutic avenues targeting different pathogenetic pathways on the salivary flow rate of SjD has been evaluated; however, most of them resulted in non-significant changes. Only a few targeted drugs have been evaluated in multiple studies, with those inducing B-cell depletion (rituximab) or exerting a combined effect on B cells and BAFF signaling (ianalumab) showing the most encouraging results regarding the salivary flow levels. Possible explanations for the common failure of single-target therapeutic approaches to restore the glandular function comprise the multifactorial nature of the disease, the simultaneous activation of overlapping innate and adaptive immune pathways, and the heterogenous cellular populations that participate in the epithelial-immune crosstalk that governs the disease pathogenesis [248,249]. Given the central role of INF-JAK-STAT axis identified through advanced molecular studies, e.g., including omics technologies, together with the dynamic interplay between SGECs and B cells, emerging targeted treatments should ideally integrate combined effects on both epithelial and immune cell components of the disease [250]. Moreover, enrollment of patients with significant diversity in clinical manifestations, as well as patients characterized by irreversible damage to salivary glands, might also influence the results regarding treatments’ effects on salivary flow rates, and symptom-based stratification of participants in clinical trials is recommended [248,250].

In conclusion, xerostomia is the clinical manifestation of the glandular parenchyma damage induced by chronic inflammatory infiltration. Residual stem cell populations within the glandular epithelium could contribute to preserving its partial secretory capacity, and their survival might represent a “critical point” prior to irreversible loss of glandular function. Therefore, stem cell-based regenerative strategies and tissue bioengineering approaches emerge as promising future interventions [249] that, when implemented prior to irreversible glandular damage, might attenuate and even prevent the disease progression.

## Data Availability

No new data were created or analyzed in this study. Data sharing is not applicable to this article.

## Abbreviations

The following abbreviations are used in this manuscript:

ABCs	Age-associated B cells
ACR–EULAR	American College for Rheumatology-European Alliance of Associations for Rheumatology
Akt	Protein kinase B
anti-SSA/Ro	anti-Sjögren’s syndrome type A
anti-SSB/La	anti-Sjögren’s syndrome type B
APRIL	proliferation inducing ligand
AQP	Aquaporin
BAFF	B-cell Activating Factor
BAFF	B-cell Activating Factor receptor
BCRs	B-cell receptors
Bregs	Regulatory B cells
BTK	Bruton’s tyrosine kinase
CTLA4	Cytotoxic T-lymphocyte-associated Protein 4
CXCL	C-X-C motif chemokine ligand
DAMPs	Danger-associated Molecular Patterns
DCs	Dendritic Cells
ESSDAI	EULAR Sjögren’s Syndrome Patient Reported Index
FasL	Fas ligand
FcRL4	Fc receptor-like 4
Gal9	Galectin 9
GLUT1	Glucose Transporter 1
GPX4	Glutathione Peroxidase 4
ICAM1	Intercellular Adhesion Molecule-1
ICOS	Inducible T-cell Co-stimulator
IFN	Interferon
IFNAR	Type I IFN receptor
Ig	Immunoglobulin
IL	Interleukin
IP3R	Inositol trisphosphate Receptors
IRFs	Interferon Regulatory Factors
JAK	Janus Kinases
LAMP3	Lysosome-associated Membrane Protein 3
LTβR	Lymphotoxin β receptor
M3R	M3 muscarinic ACh receptor
m6A	N6-methyladenosine
MAIT	Mucosal-associated Invariant T cells
MALT	Mucosa Associated Lymphoid tissue
MHC	Major Histocompatibility Complex
MSC	Mesenchymal Stem Cell
mTOR	mammalian Target of Rapamycin
mTORC1	mTOR complex 1
MZB	Marginal Zone B cells
NF-κB	Nuclear Factor-κB
NK	Natural Killer
NLRP3	NOD like receptor P3
NOD	Non-obese Diabetic
P2X7R	P2X7 receptor
PAMPs	Pathogen-associated Molecular Patterns
pDCs	plasmacytoid Dendritic Cells
PI3K	Phosphoinositide 3-kinase
poly(I:C)	polyinosinic:polycytidylic acid
pS6	Phosphorylated Ribosomal protein S6
ROS	Reactive Oxygene Species
RRM2	Ribonucleotide Reductase Regulatory Subunit M2
S1P	Sphingosine-1-P
SGECs	Salivary Gland Epithelial Cells
SjD	Sjögren’s disease
STAT	Signal Transducer and Activator of Transcription
STIM	Stromal Interaction Molecule
STING	interferon-stimulated gene
SYK	Spleen Tyrosine Kinase
TCR	T cell receptot
Tfh	T follicular helper cells
TLRs	Toll-like Receptors
TNF	Tumor Necrosis Factor
TRAIL	TNF-related apoptosis-inducing ligand
Tregs	T regulatory cells
TRPC1	Transient Receptor Potential Canonical 1
TRP	Transient Receptor Potential
VCAM	Vascular Cell Adhesion Molecule
